# Building a Digital Tool for the Adoption of the World Health Organization’s Antenatal Care Recommendations: Methodological Intersection of Evidence, Clinical Logic, and Digital Technology

**DOI:** 10.2196/16355

**Published:** 2020-10-01

**Authors:** Samira M Haddad, Renato T Souza, Jose Guilherme Cecatti, Maria Barreix, Tigest Tamrat, Carolyn Footitt, Garrett L Mehl, Inraini F Syah, Anuraj H Shankar, Özge Tunçalp

**Affiliations:** 1 Department of Obstetrics and Gynecology School of Medical Sciences University of Campinas Campinas Brazil; 2 Center for Research in Reproductive Health of Campinas (CEMICAMP) Campinas Brazil; 3 UNDP–UNFPA–UNICEF–WHO–World Bank Special Programme of Research, Development and Research Training in Human Reproduction (HRP) Department of Reproductive Health and Research World Health Organization Geneva Switzerland; 4 Ona Systems Inc Nairobi Kenya; 5 Summit Institute of Development Mataram Indonesia; 6 Centre for Tropical Medicine and Global Health Nuffield Department of Medicine University of Oxford Oxford United Kingdom; 7 Eijkman-Oxford Clinical Research Unit Eijkman Institute for Molecular Biology Jakarta Indonesia

**Keywords:** clinical decision support, antenatal care, digital health, requirements gathering, implementation, WHO guidelines, evidence-based

## Abstract

**Background:**

One of the key mandates of the World Health Organization (WHO) is to develop guidelines, defined as “a document containing recommendations for clinical practice or public health policy.” Guidelines represent the global standard for information sources shaping clinical practice and public health policies. Despite the rigorous development process and the value of guidelines for setting standards, implementing such standards within local contexts and at the point of care is a well-documented challenge. Digital technologies enable agile information management and may facilitate the adaptation of guidelines to diverse settings of health services delivery.

**Objective:**

The objective of this paper is to detail the systematic and iterative process involved in transforming the WHO Antenatal Care (ANC) guidelines into a digital decision-support and patient-record application for routine use in primary health care settings, known as the WHO digital ANC module.

**Methods:**

The WHO convened a team of clinical and digital health experts to develop the WHO digital ANC module as a tool to assist health care professionals in the implementation of WHO evidence-based recommendations for pregnant women. The WHO digital ANC module’s creation included the following steps: defining a minimum viable product (MVP), developing clinical workflows and algorithms, algorithm testing, developing a data dictionary, and the creation of a user interface or application development. The overall process of development took approximately 1 year to reach a stable prototype and to finalize the underlying content requirements of the data dictionary and decision support algorithms.

**Results:**

The first output is a reference software reflecting the generic WHO ANC guideline content, known as the WHO digital ANC module. Within it, all actionable ANC recommendations have related data fields and algorithms to confirm whether the associated task was performed. WHO recommendations that are not carried out by the health care worker are saved as pending tasks on a woman’s health record, and those that are adequately fulfilled trigger messages with positive reinforcement. The second output consists of the structured documentation of the different components which contributed to the development of the WHO digital ANC module, such as the data dictionary and clinical decision support workflows.

**Conclusions:**

This is a novel approach to facilitate the adoption and adaptation of recommendations through digital systems at the health service delivery level. It is expected that the WHO digital ANC module will support the implementation of evidence-based practices and provide information for monitoring and surveillance; however, further evidence is needed to understand how the WHO digital ANC module impacts the implementation of WHO recommendations. Further, the module’s implementation will inform the WHO’s ongoing efforts to create a pathway to adaptive and integrated (Smart) Guidelines in Digital Systems to improve health system quality, coverage, and accountability.

## Introduction

One of the key mandates of the World Health Organization (WHO) is to develop guidelines, defined as “a document containing recommendations for clinical practice or public health policy” [1]. These recommendations aim to assist policymakers and clinicians in making an informed decision on whether to undertake specific interventions based on an assessment of the anticipated impact, values and preferences, and use of resources [1]. For WHO and other institutions, such as the Institute of Medicine (IOM) and the National Institute for Health and Care Excellence (NICE), the development of guidelines entails a structured process and an underlying evidence base to support the formulation of recommendations [1,2]. As such, guidelines often represent a global standard and credible information sources for shaping clinical practice and public health policies.

Despite the rigorous development process and the value of guidelines for setting standards, applying global recommendations within local contexts and at the point of care is a well-documented challenge [2-9]. These challenges arise from both systemic issues (such as a lack of supplies, poor staff training, excessive workload, high staff turnover, lack of quality data collection, and difficulty monitoring clinical practice [7]) and barriers in accessing and interpreting recommendations during routine clinical and public health practice. Furthermore, a guideline alone is not sufficient for changing entrenched practices and often requires a combination of implementation strategies, including educational and training materials, convening conferences and meetings, auditing and providing feedback, encouraging the participation of opinion leaders, and other approaches [10]. Lastly, the variability and complexity of local conditions require significant adaptations of global guidelines, thus driving a push towards more agile, adaptive, and efficient approaches enabled by digital technologies to implement guidelines at local levels.

A complementary approach to overcoming some of the constraints associated with diffusing and incorporating static guideline recommendations into the delivery of health services is to utilize digital technologies. In particular, digital decision-support systems have emerged as an innovative mechanism to facilitate the uptake of guideline recommendations and reinforce adherence to clinical practices at the point of care [3,11-13]. Digital decision support consists of digitized job aids that can combine an individual’s health information with the health worker’s knowledge and clinical protocols to assist health workers in making diagnosis and treatment decisions [11,14,15]. Different forms of digital decision support include prompts and alerts based on a clinical algorithm, checklists according to a clinical protocol, and screening tools to identify risk and prioritize patients. Increasingly, digital decision support tools are being integrated with patient record systems to promote continuity of care and provide a more comprehensive history of the patient to bolster clinical decision-making. Despite the increasing availability of decision support systems, the accuracy of the underlying health content and adherence to the WHO (or other national bodies) guideline recommendations cannot always be verified. As such, WHO’s role in monitoring the adaptation of guidelines in digital form is a necessity.

In November 2016, the WHO published the first evidence-based guideline on antenatal care (ANC), entitled “WHO Recommendations on Antenatal Care for a Positive Pregnancy Experience” [16]. This guideline is a comprehensive document with 49 recommendations, encompassing nutritional interventions, maternal and fetal assessment, preventative interventions, common physiological symptoms, and health system interventions [17]. The recommendations ensure quality care throughout pregnancy and are based on a holistic consideration of comparative effects, values, resources, equity, acceptability, and feasibility [17].

While this guideline serves as the global standard for managing routine ANC, the guideline development team recognized that its adoption and uptake would be limited without effective implementation strategies. Considering the increasing ubiquity of mobile devices [18] and emerging uses of decision support tools, the guideline development team embarked on developing a digital decision support and health record module, which would contain the recommendations for routine use at the point of care. However, the structure of the ANC guideline, as with many other guidelines, did not readily lend itself for directly encoding into a digital system and therefore required an additional year-long process to extract the recommendations for use in a point-of-care digital system [3,6].

This paper details the systematic and iterative process involved in translating the WHO ANC guidelines into a digital decision-support and patient-record software intended for routine use in primary health care settings, hereafter referred to as the WHO digital ANC module. By describing the methods and outputs, this paper aims to offer a replicable model for distilling and optimizing guideline content for digital systems.

## Methods

### The Process

The distillation of the ANC guideline content for a digital decision-support and client-record tool required a multidisciplinary team and a series of sequential but iterative steps. These steps, detailed below, include defining a minimum viable product (MVP), developing clinical workflows and algorithms, algorithm testing, developing a data dictionary, and the creation of a user interface or application development. The overall process of development took approximately 11 months to reach a stable prototype and finalize the underlying content requirements of the data dictionary and decision support algorithms.

### Establishing Fundamental Principles

The WHO convened a team of clinical and digital health experts (henceforth called “the team”) to lead the process of developing the WHO digital ANC module. In addition to WHO staff representing maternal health and digital health expertise, the team comprised obstetrician/gynecologists from the Brazilian Center for Research in Reproductive Health of Campinas (CEMICAMP, the University of Campinas’ research arm), digital and public health–implementation experts from the SUMMIT Institute for Development, and a technology social enterprise, Ona, which is the lead technology partner developing Open Smart Register Platform (OpenSRP) open-source software. The team worked mostly virtually and held 2 in-person meetings (in November 2017 and February 2018) throughout the development process.

The team’s aim was to develop a tool that assists health care professionals in implementing WHO evidence-based recommendations when providing quality medical care to pregnant women. Initially, the team established fundamental requirements for the digital tool, which would provide decision support and a longitudinal patient record of ANC based on WHO’s evidence-based recommendations. These overarching requirements included being user-friendly and applying human-centered design principles, adaptability to a wide range of contexts (ie, based on the availability of supplies, equipment, and human resources), and the ability to monitor pregnancy progression. Further, the team sought to create a comprehensive digital tool that would be able to link with existing health and logistical management information systems (Health Management Information Systems and Logistics Management Information Systems) and local electronic medical records (EMRs). Additionally, the tool would need to generate information and monitor the health care process during the pregnancy of all women.

### Defining the Minimum Viable Product (MVP)

To set the parameters for the software’s MVP, the first iteration is designed for the primary health care (PHC) level to generate individual patient information (simple EMR) and to monitor the health care process as services are provided to all women during pregnancy. In line with the WHO recommendations for optimizing health worker roles for maternal and newborn health through task shifting, the ANC module was designed for the health worker cadre at the PHC level, namely, auxiliary nurse midwives, nurses, and midwives [19]. Future iterations, however, will be comprised of interlinked modules for community-level service provision and a patient version for women, allowing for management, feedback, and information exchange.

The first MVP provides decision support through prompts and alerts, checklists, and risk screening by health status based on WHO guidelines and clinical protocols. The WHO digital ANC module is a generic digital tool broadly applicable across all 49 ANC WHO recommendations, focusing on routine ANC (including screening and referral but not treatment for complications). Furthermore, the software documents whether required measures were implemented, and if they were not, then why not, which is important for decision-making, real-time feedback, and quality control. It is, however, customizable to diverse country contexts.

In addition to providing decision support (2.3 of WHO’s Classification of Digital Health Interventions), the module will help PHC workers identify, register, and longitudinally track pregnant women’s health status and services (2.1 & 2.2), as well as support their activity planning, scheduling (2.7), and referral coordination (2.6) [15]. Additionally, in planning for the future, the tool would be established in a way that could eventually employ machine learning algorithms, in anticipation of subsequent incorporation of artificial intelligence and predictive analytics.

### Developing Clinical Workflows and Algorithms

**Figure 1:**
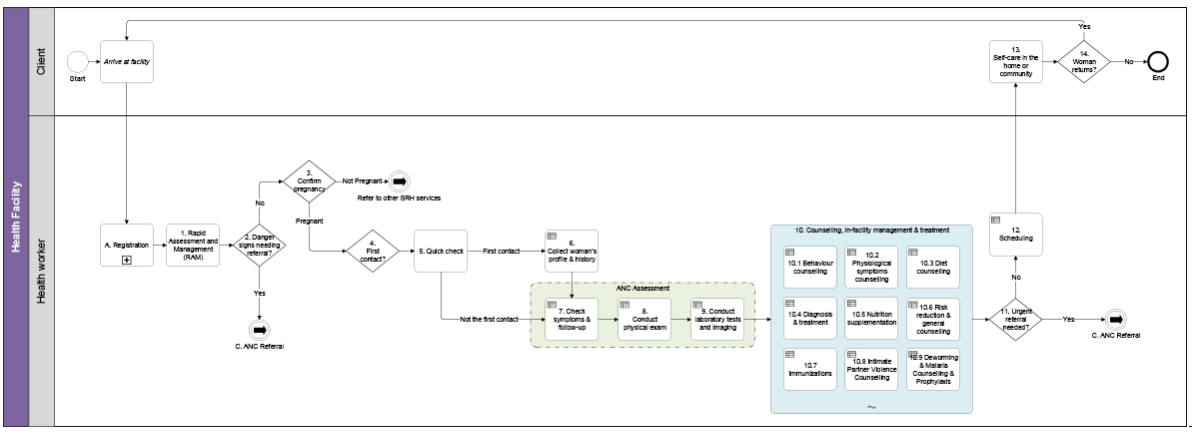
The antenatal care (ANC) contact business process. ANC: Antenatal care, SRH: sexual and reproductive health.

**Figure 2:**
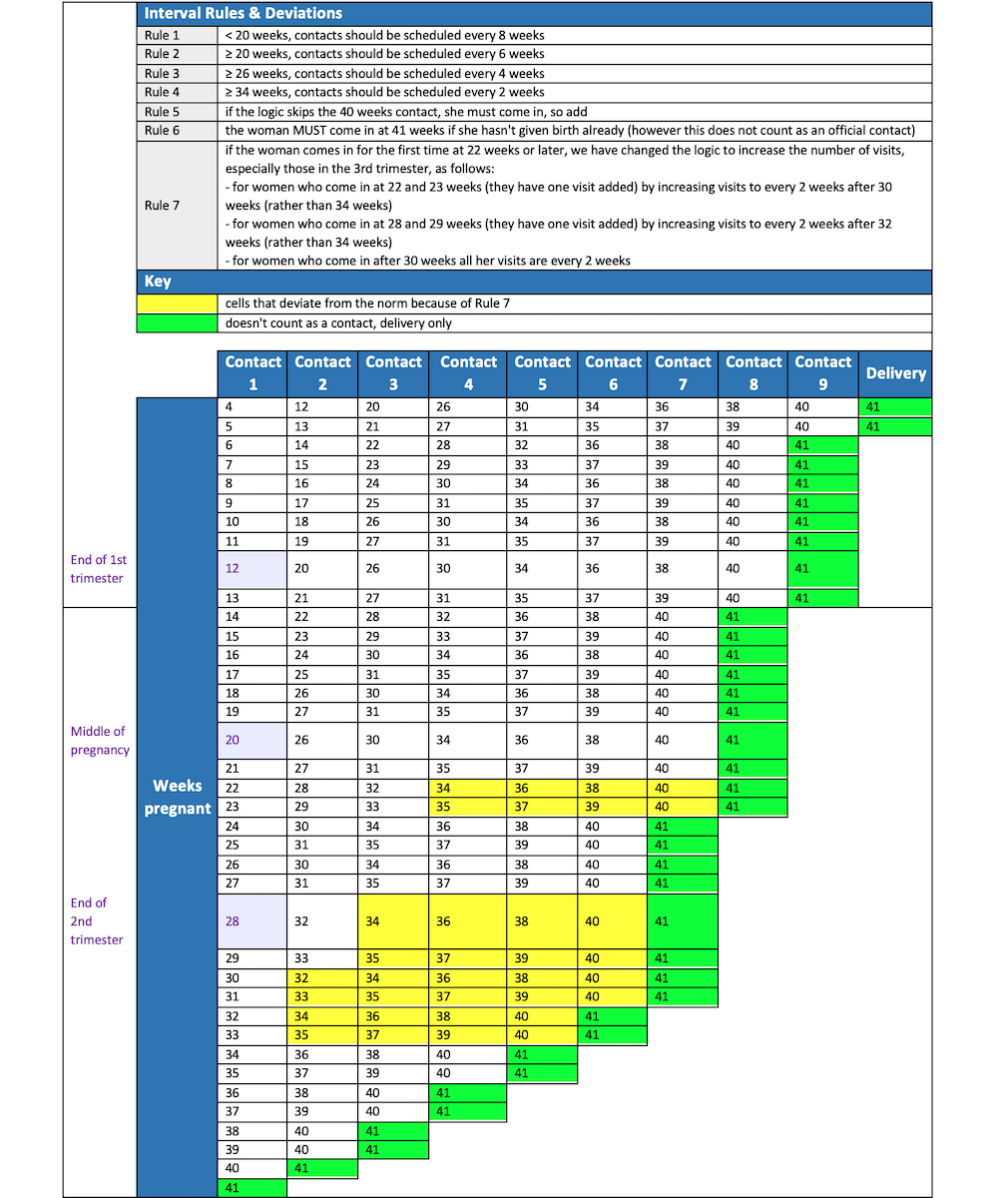
Antenatal care (ANC) 8-contact delivery schedule.

To better understand the standard patient and health worker’s actions during routine ANC contacts, the team developed relevant business processes and workflows (Figure 1) conducted by the PHC worker during ANC service delivery. The WHO’s *Pregnancy, Childbirth, Postpartum, and Newborn Care Guideline: A Guide for Essential Practice* served as a basis for this process, as it describes which procedures should be performed in clinical sequence [20]. While it is expected that each countries’ ANC workflows will vary from this generic idea, the team applied the Pareto principle, in which 80% of the content applies to all settings and 20% must be modified to local needs. Additionally, the workflows and the contact schedule (Figure 2) served as a framework for the module’s content and design.

Initially, for algorithm development, the WHO ANC recommendations were analyzed by 2 independent reviewers (SMH and RTS). Recommendations were identified, extracted, and split into spreadsheets according to their respective areas: nutritional interventions, maternal and fetal assessment, preventive measures, common physiological symptoms, and health care system interventions. Recommendations were classified in line with the ANC guideline’s categorizations: “universal” when they could be implemented in any scenario; “context-specific” when characteristics of the population were necessary to trigger the actions; “context-specific research” when further studies were needed to formalize the recommendation; and “not recommended” when the action should be avoided due to a lack of a sufficient scientific basis to support its performance.

Upon further review, the team removed the “context-specific research” recommendations from the MVP.

**Figure 3:**
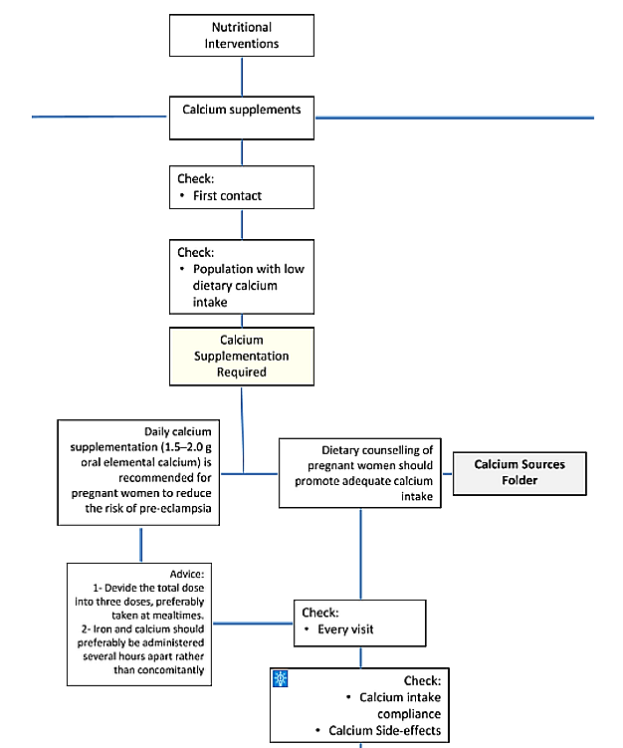
Example of the clinical algorithm: calcium supplementation recommendation.

Other official WHO guidelines were consulted for clarification or for additional recommendations considered important for routine ANC (ie, influenza vaccination, pre-eclampsia screening, and hepatitis B and C testing [21-24]). When necessary, some adaptations were made to align with the clinical logic of the ANC service delivery process. These suggestions were assessed and agreed upon by the team. Decision trees were then created for each of the ANC and additional recommendations, considering parameters that trigger action during an ANC contact (for example, calcium supplementation after 20 weeks gestation in women at high-risk for pre-eclampsia). Decision trees were also shaped by population-level characteristics (eg, folic acid and elemental iron supplementation according to the prevalence of anemia in the population), the local availability of health care equipment (eg, type of urine or syphilis tests depending on the laboratory facilities), and individual-level variables (eg, hepatitis C testing, if injectable drug user). Thus, a single algorithm structure or decision tree was developed to take all of these elements into consideration (Figure 3).

### Algorithm Testing

**Figure 4:**
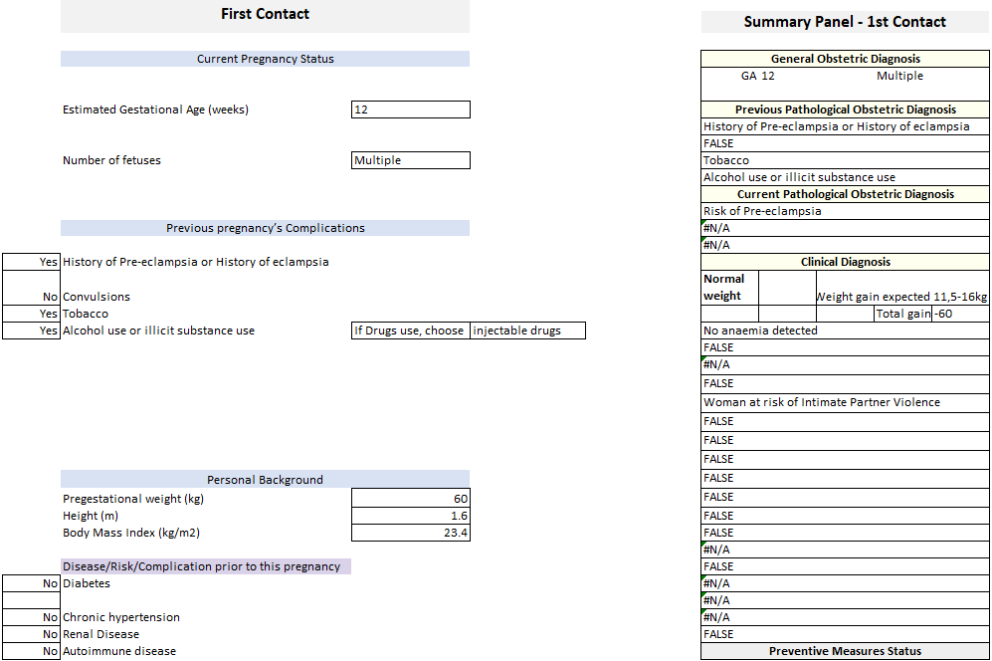
Excel testing form.

Prior to the software’s development, a test version was constructed (by SMH) using Excel resources that included diverse functions linking variables across different worksheets. The first version of the system design was outlined in the Excel testing form, creating an interactive spreadsheet (Figure 4). The testing form displayed real-time clinical diagnoses, the ANC recommendations, and additional guidance on the health care process, all automatically elaborated by the system during data entry and guided by the logic underlying the clinical algorithms. Initial tests were performed using fabricated patient cases that explored data relative to all algorithms, probing all decision trees. The logic of the algorithm was thus reviewed and corrected, where necessary. Furthermore, the Excel prototype was helpful to visualize how the system could interact with the user in real time as a tool for support in clinical decisions in ANC.

### Development of the Data Dictionary

After a detailed review of the algorithms, the data elements which comprised them were separated into sections, including population and local setting characteristics, socio-demographic data, information on current pregnancy, medical (including immunization) and obstetric history, lifestyle and habits, physiological symptoms of pregnancy, physical examination and laboratory tests, counseling, and treatment. Initially, only data elements required to trigger recommendation-related algorithms were included in the data entry forms. As an example, basic variables for the early identification of sexually transmitted infections (STIs) were included, without further development of the complete algorithm for the differential diagnosis and management of these conditions (except syphilis and HIV, which are included in the ANC guidelines). For STIs as well as other issues, algorithms allow for the health worker to identify risk and subsequently support referral for further investigation or treatment.

**Figure 5:**
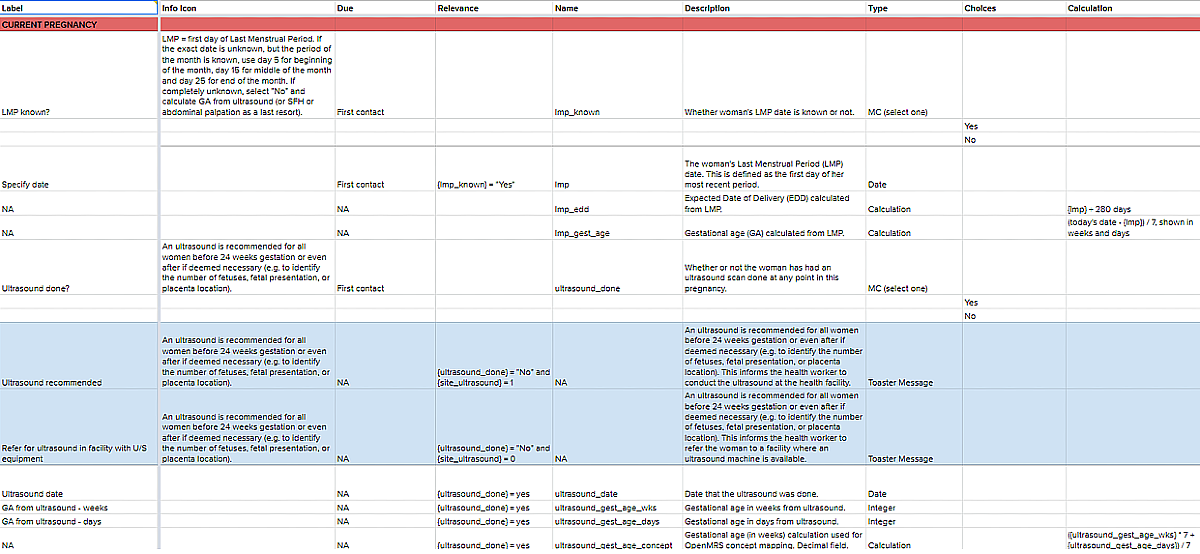
Data Dictionary - Profile Tab. ANC: antenatal care; GA: gestational age; HIV: human immunodeficiency viruses, LMP: last menstrual period; MC: multiple choice; NA: not applicable; SFH: symphysis fundal height; STI: sexually transmitted infection.

From these efforts, the team constructed the data dictionary, which is a shared Google spreadsheet that contains all the clinical content and decision-support logic to be used to create the data entry forms and decision-support workflows in the WHO digital ANC module (Figure 5). At this stage, the team also incorporated additional content in the data dictionary for the complete provision of routine ANC (eg, fetal heart rate, vaginal examination, maternal pulse, blood pressure, and temperature measurement), which are assumed standard clinical practices and therefore are not part of the ANC guidelines. The data dictionary served as the primary technical requirement for the software developers to build the MVP.

Additionally, all the variables in the data dictionary were mapped to standardized medical terminology codes, such as the International Classification of Diseases (ICD), Logical Observation Identifiers Names and Codes (LOINC), and Systematized Nomenclature of Medicine (SNOMED). Thus, the team also sought to standardize terminologies to ensure interoperability and ease of data aggregation for future integration with databases. For clinical care, terminologies are structured vocabularies covering health related concepts, such as diseases, diagnoses, laboratory tests, and treatments, to enable the storage, analysis, and exchange of data in a consistent and standard way [25,26].

### Creation of User Interface and Application Development

As content was being developed in the data dictionary, the team also began creating mockups of the module's user interfaces, which allowed team members to collaboratively discuss, iterate on, and come to a consensus on the design and workflows required for the MVP. As the data dictionary content was completed over time, the team was able to enrich the interface designs with plausible clinical content, which further allowed for testing the designs with additional collaborators, including medically trained colleagues at the WHO and the Center for Research in Reproductive Health of Campinas (CEMICAMP), to elicit better feedback. During this iterative process, mockups were shared and discussed on Marvel [27]. After several rounds of feedback and iteration, the data dictionary and mockups were approved, which allowed the software development of the module to begin building it.

## Results

### Outputs

The methods described above resulted in 2 outputs. The first output is a reference software reflecting the generic WHO ANC guideline content, known as the WHO digital ANC module. The second output consists of the structured documentation of the different components which contributed to the development of the WHO digital ANC module, such as the data dictionary, clinical decision-support workflows, and mockups.

### WHO Digital ANC Module

**Figure 6:**
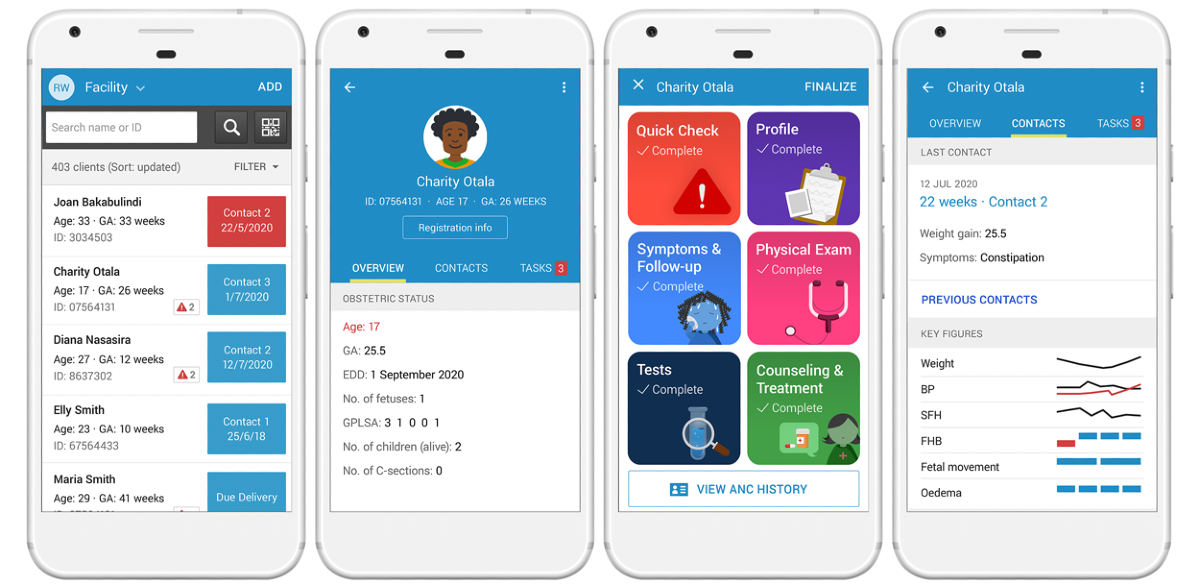
Screenshots of the World Health Organization Digital Antenatal Care (WHO digital ANC) module. A: List of patients; B: Individual patient record summary; C: Home screen; D: Patient contact summary.

The current version (1.0) of the WHO digital ANC module allows health workers to manage their patients’ records as well as filter for women diagnosed with certain conditions, view attention flags, easily identify women overdue for contacts, and sort entries for gestational age, due date, and first and last name (Figure 6A). Health workers can also see a summary of a woman’s prior contacts and any attention flags; prompts noting conditions for close follow-up (eg, advanced maternal age, twin pregnancy) are highlighted in red (Figure 6B). For further details, all the information from the woman’s prior contacts can also be accessed in the profile overview.

During a contact, the health worker can access the landing page (Figure 6C), which groups data entry forms based on the major clinical workflows that were identified during the design phase: Quick Check, Profile, Symptoms & Follow Up, Physical Exam, Tests, and Counselling & Treatment. Within these “containers,” all ANC recommendations have a related question to record whether the health worker has performed the associated task. WHO recommendations that are not carried out are placed on a pending list of tasks, and those that are adequately fulfilled prompt a message with positive reinforcement. Other highlighted features include the following:

- Alerts to remind the health worker of a recommended action (eg, “An ultrasound is recommended for all women before 24-weeks gestation or even after if deemed necessary, to identify the number of fetuses, fetal presentation, or placenta location”), to provide positive reinforcement (eg, “Woman is fully immunized against tetanus!”), or to flag a risk (eg, “Pre-eclampsia risk counseling: the use of aspirin after 12 weeks gestation is recommended as well as calcium if low dietary intake area. Please also provide counseling”)
- A task list of summarized actions to allow the health worker to identify potentially missed opportunities at the end of the contact, or to be reminded of pending actions (eg, test results that need to be recorded)
- Sparklines depicting trends in certain data elements (eg, weight, blood pressure, symphysis fundal height) (Figure 6D)
- Additional informative materials for health workers regarding nutrition counseling

In addition, the MVP of the module also contains an interface for the manager at the health facility to set population levels (such as HIV prevalence) and site infrastructure parameters (such as the availability of ultrasound machines). For communication with the pregnant women, the MVP allows for the sending of SMS text messages (if the pregnant woman consents), and in a future version, there would be a complete interface sharing information as well as a virtual “pregnant woman card.” Finally, the MVP seeks to increase the use of data for accountability to drive quality service provision through the automated aggregation of key indicators and to increase the availability of dashboards for use by different stakeholders such as program managers.

### Test Cases

In order to assess the module’s components, 2 researchers (SMH and RS) elaborated 67 clinical test cases. The cases were designed to encompass the largest number of possible scenarios, testing all clinical algorithms. An online database was created using Google Forms, incorporating variables from the data dictionary. The team tested these cases to check the decision-support logic in the module and worked with the software developer to correct any issues discovered.

### Structured Documentation

**Figure 7:**
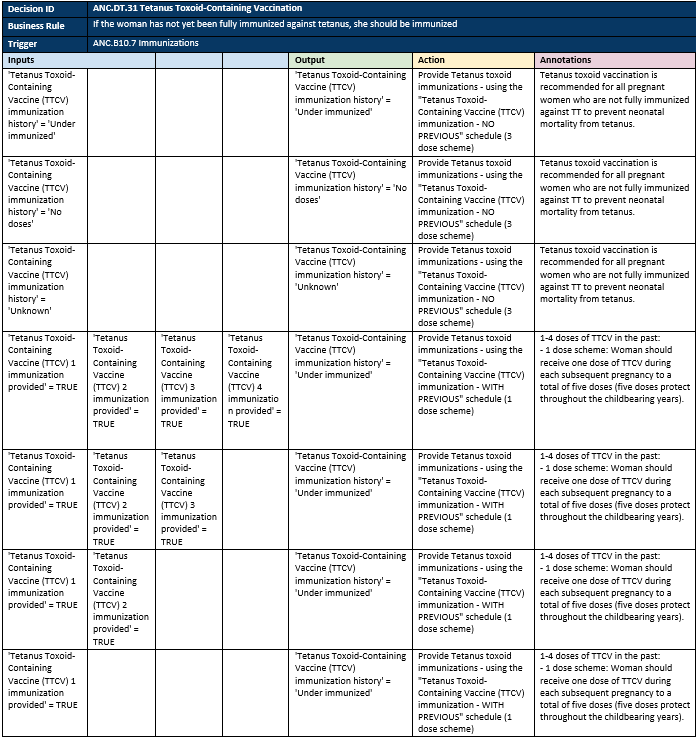
Example decision-support table.

Throughout the WHO digital ANC module’s development, the team meticulously documented the process for its creation. Firstly, during the definition of the MVP, targeted user personas were defined (PHC workers) and user stories were created (role-play of a typical ANC contact). Secondly, the aforementioned business processes, workflows, and contact schedule set the parameters for the module’s content (Figures 1 and 2). Next, the data dictionary aided in creating the core data elements needed for the ANC service provision (Multimedia Appendix 1). The data elements linked together through the algorithms (based on the decision trees) and were also elaborated further in decision-support tables (Figure 7). Finally, the module provides a link between an individual’s data elements to the components (numerators and denominators), which constitute established ANC indicators to simplify the data aggregation process.

Beyond making the WHO digital ANC module available as a digital decision-support and client-record tool within OpenSRP, WHO is also making the structured documentation publicly available as digital accelerator kits [28]. This structured documentation is independent of the OpenSRP software and intended to accompany the WHO digital ANC module to assist countries in understanding the underlying data elements and algorithms to facilitate the customization of the ANC module to their context. Additionally, countries that may already have digital systems in place will be able to use the structured documentation (eg, the data dictionary and decision tables) to ensure that the content is aligned with the WHO guidelines, or at least to have a starting point from which they can design the digital system if they choose to use a separate software platform.

These different components of the structured documentation were derived from standardized methodologies in health informatics, most notably, business process mapping notation (BPMN) for the workflows, development of the data dictionary (which is itself a derivative of the XLS form standard), and decision model notation (DMN) for the decision logic. The formulation of the kits´ components was documented by the team, and the process of releasing structured documentation for digital systems is also being replicated across other health domains (eg, family planning, HIV).

## Discussion

There is a growing recognition of the multitude of software applications targeted at health workers [29,30]; however, the WHO digital ANC module reflects a systematic effort to ensure that the latest WHO clinical guidelines are available in digital form for routine use. As such, the distinguishing factor of the WHO digital ANC module is not necessarily the digital functionalities but rather the availability of the 2016 WHO ANC guideline content encoded in a format that countries can readily adapt and incorporate into service delivery [3].

Challenges in translating clinical guideline content to digital systems include potential misinterpretations that may result from not having the precise logic and data elements within the guideline itself [3,31]. Furthermore, software development companies building digital decision support tools are often required to start from scratch in translating guideline content to digital workflows. With the creation of the WHO digital ANC module and accompanying structured documentation, the challenges associated with misinterpretation of the guideline content or unnecessary replication in distilling the recommendations are thus minimized. In settings where there are no digital systems for routine ANC in place, countries can leverage the WHO digital ANC module as an out-of-the-box generic digital system that can then be easily adapted to their local contexts instead of starting from scratch. Alternatively, in settings where electronic medical records and other digital systems already exist, the structured documentation in the WHO’s forthcoming digital accelerator kits can be used to inform the content within the digital system. Additionally, the ANC digital accelerator kit (upon which the module is based) aims to simplify and standardize individual-level and aggregate data collection, a key component to tracking guideline implementation at national and global levels.

The development of the WHO digital ANC module also resonates with an established approach developed by Boxawala et al [3] to manage the translation of narrative guideline recommendations to digital decision support tools. This framework consists of 4 sequential knowledge layers: the unstructured narrative recommendation as written in the guideline (Layer 1); a semi-structured derivative of the guideline recommendation in the form of decision trees, flowcharts, and data logic (Layer 2); a computable form of the recommendation represented in software code (Layer 3); and an executable digital decision-support tool that can be used directly by health workers (Layer 4) [3]. In our application of this framework, the WHO ANC recommendations represent Layer 1, the structured documentation of the digital accelerator kit corresponds to Layer 2, and the ANC module consists of Layer 3. Currently, Layer 4 is under development and seeks to translate the ANC recommendations into a computable and coded format that can be directly ingested by a digital decision-support system. This includes Fast Healthcare Interoperability Resources (FHIR), which will be embedded into the ANC module. This will facilitate the export of data in a standardized way for analysis by CQL and other methods optimized for FHIR, increasing interoperability.

Following the completion of the module, the team conducted user testing of the module with nurses and midwives in Indonesia and physicians in Brazil. These professionals were not part of the previous stages of the module’s creation. A standardized protocol was developed to train health workers to use the module and elicit feedback from these health workers through focus group discussions and in-depth interviews. From these experiences, feedback was compiled, and the team made changes where deemed necessary prior to the generic version 1.0’s release. The WHO digital ANC module (version 1.0) and underlying standardized documentation will be released for global use to support countries and health care providers to adapt and adopt the WHO ANC recommendations.

We believe that this first version of the WHO digital ANC module is robust; however, future efforts are currently underway to support its implementation in various countries. As part of this process, the team is developing training materials (videos, handbooks, presentations, etc) for health workers at primary health centers. Additionally, a framework and indicators for monitoring, evaluating, and supervising the module’s implementation and its impact on health worker performance will be created [32-34]. It is expected that the WHO digital ANC module will support the implementation of evidence-based practices and provide information for monitoring and surveillance; however, further evidence is needed to understand how the ANC module impacts the implementation of the WHO recommendations. The WHO is developing an implementation research study to assess whether the use of the module improves the fidelity to WHO ANC recommendations in various countries.

Ultimately, prescriptive and rigid recommendations are not appropriate or optimal, and over time, the WHO has moved toward context-specific recommendations and has developed tools that allow for the design of health care services based on local settings [35]. However, at the same time, doing so has also changed the complexity of interpreting and implementing recommendations. Digital tools have the potential to facilitate and accelerate the implementation of such guidelines on the ground. The WHO digital ANC module is a pioneering effort to create a roadmap for that process, which will inevitably be needed for policies across other health domains. The adaptive processes required for the implementation of the WHO ANC recommendations were enhanced at each step in the development of the WHO digital ANC module. This is a novel approach to facilitating the adoption and adaptation of guidelines through digital systems at the health delivery service level. The WHO will release the module for countries’ adaptation and use. Further, the module’s implementation will inform the WHO’s ongoing efforts to create a pathway to adaptive and integrated (Smart) Guidelines in Digital Systems to improve health system quality, coverage, and accountability.

## Appendix

Multimedia Appendix 1 Overview of data element categories.

## Abbreviations

ANC: antenatal care
EMR: electronic medical record
FHIR: Fast Healthcare Interoperability Resources
MVP: minimum viable product
OpenSRP: Open Smart Register Platform open-source software
PHC: primary health care
STI: sexually transmitted infection
WHO: World Health Organization
