# Correction: Building a Digital Tool for the Adoption of the World Health Organization’s Antenatal Care Recommendations: Methodological Intersection of Evidence, Clinical Logic, and Digital Technology

**DOI:** 10.2196/24891

**Published:** 2020-10-13

**Authors:** Samira M Haddad, Renato T Souza, Jose Guilherme Cecatti, Maria Barreix, Tigest Tamrat, Carolyn Footitt, Garrett L Mehl, Inraini F Syah, Anuraj H Shankar, Özge Tunçalp

**Affiliations:** 1 Department of Obstetrics and Gynecology School of Medical Sciences University of Campinas Campinas Brazil; 2 Center for Research in Reproductive Health of Campinas (CEMICAMP) Campinas Brazil; 3 UNDP–UNFPA–UNICEF–WHO–World Bank Special Programme of Research, Development and Research Training in Human Reproduction (HRP) Department of Reproductive Health and Research World Health Organization Geneva Switzerland; 4 Ona Systems Inc Nairobi Kenya; 5 Summit Institute of Development Mataram Indonesia; 6 Centre for Tropical Medicine and Global Health Nuffield Department of Medicine University of Oxford Oxford United Kingdom; 7 Eijkman-Oxford Clinical Research Unit Eijkman Institute for Molecular Biology Jakarta Indonesia

In “Building a Digital Tool for the Adoption of the World Health Organization’s Antenatal Care Recommendations: Methodological Intersection of Evidence, Clinical Logic, and Digital Technology” (J Med Internet Res 2020;22(10):e16355) the authors noted three errors.

The name of author Tigest Tamrat was incorrectly listed as “Tigist Tamrat”. This has now been changed to the correct spelling.

Under the section "Structured Documentation", one instance of the phrase "business process mapping (BPM)" has been corrected to "business process mapping notation (BPMN)".

Figure 6 has been replaced with a new version of subsection B (seen below), as the authors did not have permission to use images displayed in the originally published version of subsection B. Figure 6 subsections A, C, and D have not been changed from the originally published version.

**Figure 6:**
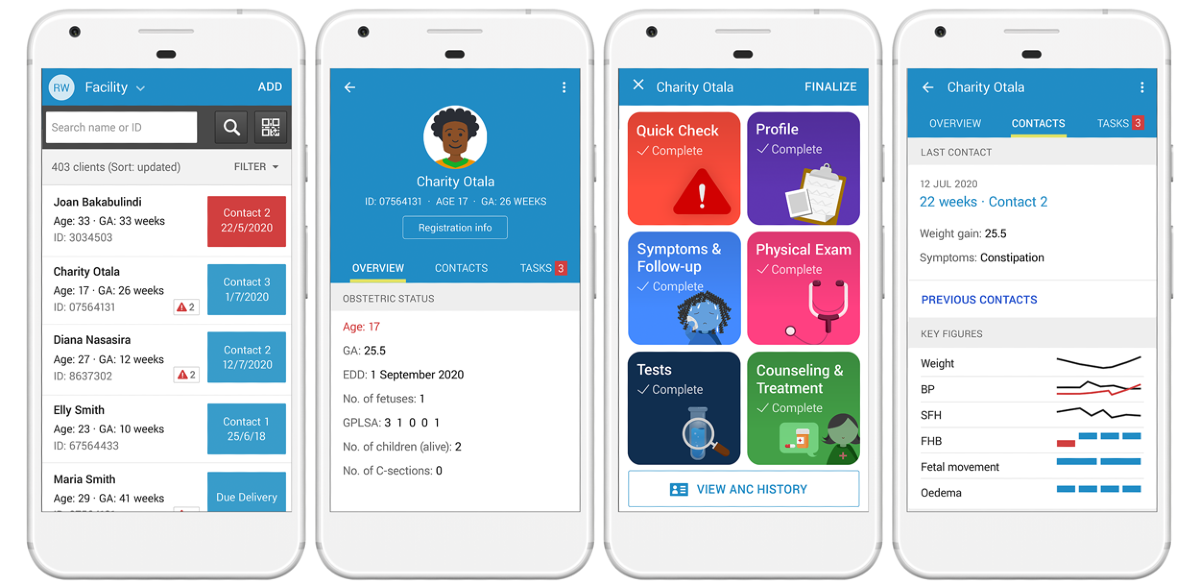
Screenshots of the World Health Organization Digital Antenatal Care (WHO digital ANC) module. A: List of patients; B: Individual patient record summary; C: Home screen; D: Patient contact summary.

The correction will appear in the online version of the paper on the JMIR Publications website on October 13, 2020, together with the publication of this correction notice. Because this was made after submission to PubMed, PubMed Central, and other full-text repositories, the corrected article has also been resubmitted to those repositories.

