# Supplementary material for: Building a Digital Tool for the Adoption of the World Health Organization’s Antenatal Care Recommendations: Methodological Intersection of Evidence, Clinical Logic, and Digital Technology
Source: J Med Internet Res. 2020 Oct 1;22(10):e16355. doi: 10.2196/16355 (PMC7983224; doi:10.2196/16355)
Supplement: Multimedia Appendix 1 [file jmir_v22i10e16355_app1.docx]

**Multimedia Appendix 1: Overview of data element categories.**

| **Workflow activity ID** | **Data Element Name** | **Description** |
| --- | --- | --- |
| **B1. Arrive at clinic** | N/A |  |
| **B2. Registration** | B2.1 ID number | Identifier for pregnant woman (could be national ID or other form of unique ID) |
|  | B2.2 First name | Woman's first name. |
|  | B2.3 Surname | Woman's family or last name. |
|  | B2.4 Date of birth (DOB) | Woman's DOB. |
|  | B2.5 Age | Age in years calculated from DOB. |
|  | B2.6 Address | Woman's home address. |
|  | B2.7 Mobile phone number *(optional)* | Woman's mobile phone number *(optional)* |
|  | B2.8 Does she want to receive reminders during pregnancy? *(optional)* | Whether or not the woman wants to receive reminders regarding her ANC contacts and health status during pregnancy. *(optional)* |
|  | B2.9 Alternate contact name | Name of an alternate contact for the woman (e.g. partner, husband, mother, etc.) |
|  | B2.10 Alternate contact phone number | Phone number of the alternate contact. |
| **B3. Quick check** | B3.1 Reason for coming to facility | Reason why the woman came to health facility, whether it was a scheduled visit or based on a complaint. |
|  | B3.2 Specific complaint(s) | If the woman came to the facility with a specific complaint, select the complaint(s) from the list. |
|  | B3.3 Danger signs | Determine if a woman has any danger signs, based on multiple choice options. |
|  | B3.4 How to proceed with danger signs | How danger sign was managed, based on whether there was a referral or if health worker proceeded to complete the contact. |
| **B4. Danger signs requiring referral** | | |
| **B5. Urgent referral** | | |
| **B6. Determine if first contact** | | |
| **B7. ANC profile and history** | B7.1 Highest level of school | Highest level of school the woman has reached |
|  | B7.2 Occupation | Woman's occupation |
|  | B7.3 Date of Last Menstrual Period (LMP) | Date of first day of Last Menstrual Period(LMP) |
|  | B7.4 Ultrasound (U/S) date | Date the ultrasound was done. |
|  | B7.5 GA from ultrasound - weeks | Gestational age (GA)  (weeks only) from the ultrasound results. |
|  | B7.6 GA from ultrasound - days | Gestational age (GA)  (days only) from the ultrasound results. |
|  | B7.7 GA from SFH or abdominal palpation - weeks | If LMP is unknown and ultrasound wasn't done or it wasn't done early (before 24 weeks), then assess GA based on Symphysis Fundal Height (SFH) or abdominal palpation. |
|  | B7.8 Preferred gestational age | Preferred gestational age based on option of LMP; ultrasound; SFH or abdominal palpation |
|  | B7.9 No. of pregnancies | Total number of times the woman has been pregnant (including this pregnancy). |
|  | B7.10 No. of pregnancies lost/ended | Total number of miscarriages and abortions (before 22 weeks / 5 months) |
|  | B7.11 No. of live births | Total number of live births (after 22 weeks) |
|  | B7.12 Preterm birth | Whether last live birth preterm (<37 weeks)? |
|  | B7.13 No. of stillbirths | Total number of stillbirths (after 22 weeks) |
|  | B7.14 No. of C-sections | Total number of C-sections. |
|  | B7.15 Past pregnancy complications | Woman’s history of complications in any previous pregnancy. |
|  | B7.16 Allergies | Woman’s history of allergies |
|  | B7.17 Gynecological procedures | Woman’s past surgeries and gynecological procedures |
|  | B7.18 Health condition | Woman’s chronic and past health conditions, including HIV, diabetes, hypertension. |
|  | B7.19 Immunization status | Woman’s history of past vaccinations, including TT, Hep B, and influenza. |
|  | B7.20 Medications | Medications the woman is taking (can be selected from drop-down list) |
|  | B7.21 Caffeine intake | Woman’s caffeine intake. |
|  | B7.22 Tobacco use | Woman’s tobacco use and exposure to second-hand smoke. |
|  | B7.23 Condom use | Woman’s (and her partner’s) use of condoms during sex |
|  | B7.24 Alcohol/substance use | Woman’s consumption of alcohol or use of substances. |
|  | B7.25 Partner HIV status | Select if partner HIV status is known as Positive or Negative, or if unknown |
| **B8. Symptoms and follow-up** | B8.1 Medication follow-up | What medications (including supplements and vitamins) is the woman still taking? |
|  | B8.2 Calcium supplement follow-up | Is the woman continuing to take calcium supplements? |
|  | B8.3 IFA tablets follow-up | Is the woman continuing to take the IFA supplements and if she is experiencing side effects to the IFA. |
|  | B8.4 Aspirin tablets follow-up | Is the woman continuing to take aspirin tablets? |
|  | B8.5 Vit-A supplement follow-up | Is the woman continuing to take Vitamin A supplements? |
|  | B8.6 Penicillin treatment for syphilis | Is the woman continuing to take penicillin treatment for syphilis? (If appropriate) |
|  | B8.7 Reported behavior follow-up | Do the reported behaviors persist, including caffeine, tobacco use, exposure to second-hand smoke, condom use during sex, alcohol or substance use? |
|  | B8.8 Reported physiological symptoms follow-up | Do physiological symptoms (e.g. nausea, heartburn, low back pain,  pelvic pain, varicose veins, oedema) persist? |
| **B9. Physical exam** | B9.1 Height | Woman's height in centimetres. |
|  | B9.2 Pre-gestational weight | Woman's pre-gestational weight in kilograms. |
|  | B9.3 Current weight | Woman's current weight in kilograms from this contact. |
|  | B9.4 Blood pressure | Woman's systolic and diastolic blood pressure in mmHg. |
|  | B9.5 Temperature | Woman's body temperature in degrees Celsius. |
|  | B9.6 Pulse | Woman's pulse rate in beats per minute. |
|  | B9.7 Pallor | Whether or not the woman has pallor. |
|  | B9.8 Respiratory exam | Woman's respiratory condition. |
|  | B9.9 Cardiac exam | Woman's cardiac condition. |
|  | B9.10 Breast exam | Whether or not the result of the breast exam is normal. |
|  | B9.11 Abdominal exam | Result of the abdominal exam, and whether normal, abnormal, or not done. |
|  | B0.12 Pelvic exam | Result of the pelvic exam, and whether normal, abnormal, or not done. |
|  | B9.13 Cervical exam | Whether or not the health worker performed a cervical exam, and centimeters dilated. |
|  | B9.14 Oedema present | Whether or not the woman has oedema, and if so the type of oedema and severity |
|  | B9.15 Fetal movement | Whether or not the health worker observes fetal movement. |
|  | B9.16 Fetal heartbeat | Whether or not the health worker observes a fetal heartbeat, and heart rate in beats per minute. |
|  | B9.17 Number of fetuses | Number of fetuses the woman is carrying. |
|  | B9.18 Fetal presentation | Presentation of the fetus in the uterus. |
| **B10. Laboratory and imaging tests** | B10.1 Ultrasound | Whether an ultrasound was done, or  the date of the ultrasound and gestational age. |
|  | B10.2 Blood type | Whether a blood type test was done, or the date of the blood test, the woman's blood type and Rh factor. |
|  | B10.3 HIV test | Whether an HIV test was done, or the date of the HIV test and result |
|  | B10.4 Partner HIV | Whether partners’ HIV test was done/is known, or the date of the HIV test and result |
|  | B10.5 Hepatitis B | Whether Hepatitis B test was done, type of test, the date of the Hep B test, and result |
|  | B10.6 Hepatitis C | Whether Hepatitis C test was done, type of test, the date of the Hep C test, and result |
|  | B10.7 Syphilis | Whether syphilis test was done, type of test, the date of the syphilis test, and result |
|  | B10.8 Urine | Whether urine test was done, type of test, the date of the urine test, and result |
|  | B10.9 Blood glucose | Whether blood glucose test was done, type of test, the date of the test, and results |
|  | B10.10 Blood hemoglobin | Whether blood hemoglobin test was done, type of test, the date of the test, and results |
|  | B10.11 Tuberculosis (TB) screening | Whether TB test was done, the date of the test, and results |
| **B11. Counselling and in-facility treatment** | | |
| **B12. Urgent referral** | | |
| **B13. Scheduling follow-up visit** | | |
